# Cup-to-disc ratio measured clinically and via OCT in pediatric patients being monitored as glaucoma suspects for suspicious optic discs

**DOI:** 10.3389/fopht.2024.1479286

**Published:** 2024-12-03

**Authors:** Caroline Maria Zimmermann, Nur Cardakli, Courtney Lynn Kraus

**Affiliations:** ^1^ Department of Ophthalmology and Visual Sciences, University of Maryland School of Medicine, Baltimore, MD, United States; ^2^ Wilmer Eye Institute, Johns Hopkins University School of Medicine, Baltimore, MD, United States

**Keywords:** cup-to-disc ratio, glaucoma suspect, pediatric glaucoma, optical coherence tomography, pediatric

## Abstract

**Purpose:**

Compare cup-to-disc ratio (CDR) measured by clinical assessment and optical coherence tomography (OCT) in pediatric eyes being monitored as glaucoma suspects for suspicious optic disc appearance.

**Design:**

Retrospective cross-sectional study.

**Methods:**

An institutional study following 221 eyes from 122 unique pediatric glaucoma suspects being monitored due to increased or asymmetric appearance of CDR. Ophthalmologic findings, including visual acuity, intraocular pressure, CDR measured by clinical assessment, average retinal nerve fiber layer thickness, and average CDR measured by OCT, were recorded for each participant’s initial and final examinations. CDRs measured clinically and by OCT were compared at both initial and final presentations.

**Results:**

Average age at presentation was 9.0 years old (95% CI: 8.0-9.9), and mean length of follow-up was 5.0 years (95% CI: 5.4-4.5). At initial presentation, 53 eyes had CDRs recorded by both clinical assessment and OCT, and at final presentation, 93 eyes had CDRs measured by both modalities. CDR measured by OCT was significantly larger than CDR measured clinically on initial and final presentation (p=0.002, p<0.001).

**Conclusions:**

Measurements of CDR by clinician assessment were significantly smaller than measurements obtained via OCT imaging. However, the average difference between CDR measured clinically and by OCT was <0.1. Thus, OCT may be a suitable way to measure CDR in pediatric glaucoma suspects, especially when clinical exam proves difficult. Further research is needed to assess CDR in glaucoma suspects using OCT longitudinally and in the context of other optic disc measurements, such as disc area.

## Introduction

Glaucoma describes a group of diseases that involve damage to the optic nerve (1). Pediatric patients can develop glaucoma as a primary congenital or juvenile disease or secondary to another ocular or systemic disease (2). Because glaucoma may lead to significant visual impairment if left untreated, early diagnosis is key to preventing permanent vision loss. Glaucoma is often associated with increased intraocular pressure (IOP) and cup-to-disc ratio (CDR), and measurements and assessments of IOP and CDR are essential in screening and monitoring these patients. Signs of glaucomatous optic neuropathy, such as increased CDR or asymmetry of the CDR between two eyes, raise suspicion for glaucoma and may necessitate more frequent monitoring for progression of disease (3). Suspicious CDR findings, however, may also be physiologic and never progress to pathologic disease. Certain optic nerve findings, such as a large optic disc or a small vertical CDR, are more associated with physiological cupping than development of glaucoma (4).

Studies have shown that clinical assessments of CDR can be imprecise and have interobserver variability, even among glaucoma experts (5, 6). Optical coherence tomography (OCT) is an imaging tool that can measure CDR and has been shown to provide objective and reproducible measurements (7, 8). Previous studies have found that CDR measurements by OCT are larger than measurements made by direct ophthalmoscopy in adults (9, 10). Thus, CDR measurements by clinical assessment may be underestimated. In children, achieving an adequate view of the optic nerve during clinical examination can be difficult due to patient cooperation and needs to rely more frequently on indirect ophthalmoscopy with lenses providing less magnification. Therefore, the addition of imaging modalities to nerve assessment may provide a valuable way to evaluate the risk of progression to glaucoma among pediatric glaucoma suspects. Previous studies have utilized various imaging modalities, including OCT and fundus photography, to evaluate the optic disc in children (11–13). Additionally, advancements in technology, like handheld and intraoperative OCT, are beginning to address some of the difficulties of using OCT in the pediatric population (14). This paper aims to report the relationship between optic nerve evaluation by OCT and clinical assessment of CDRs in a cohort of pediatric glaucoma suspects being monitored for suspicious optic nerve appearance.

## Materials and methods

Pediatric patients with a suspicious appearance of the optic disc were selected as a subgroup from a larger study of pediatric glaucoma suspects conducted by our group (15). Potential cases were identified through retrospective chart review using ICD9/ICD10 codes for “physiologic cupping” (H47.239), “glaucoma suspect” (H40.00, H40.01), and “ocular hypertension” (H40.05). Additional cases were identified using codes for ocular and systemic conditions associated with an increased risk of glaucoma, including neurofibromatosis 1, Axenfeld-Rieger, Sturge Weber syndrome, Peters anomaly, aphakia/pseudophakia, trauma, hyphema, aniridia, and anterior segment dysgenesis. Eyes being followed as glaucoma suspects for suspicious disc appearance in the absence of ocular hypertension were included in this subset of patients being monitored as “suspects for suspicious disc appearance” and are reported here. Suspicious CDRs were determined using criteria set by the Childhood Glaucoma Research Network (CGRN; CDR ≥ 0.5, asymmetry of CDR ≥ 0.2 between two eyes, or notching or narrowing of the neuroretinal rim) (3). All patients were seen at the Johns Hopkins Wilmer Eye Institute for an initial visit between January 1, 2005 and June 1, 2016, were 20 years old or younger at initial presentation, and had a minimum follow-up of at least 180 days from initial evaluation. The study was approved by the Johns Hopkins Institutional Review Board prospectively and abided by the Declaration of Helsinki.

Demographic data, including age, sex, and race/ethnicity, were collected for study participants via retrospective chart review. Ophthalmologic findings, including visual acuity (VA), IOP, CDR measured by clinical assessment, average retinal nerve fiber layer (RNFL) thickness, RNFL thickness in the superior, inferior, nasal, and temporal quadrants, and average CDR measured by OCT, were recorded for each participant at both initial presentation and last follow-up visit at time of data collection. When possible, date of initial exam matched the date of initial OCT and date of last follow-up exam recorded matched the date of last OCT recorded. When the date of OCT testing did not match the date of clinical exam, the closest OCT testing date was recorded. Clinically measured CDR on the date closest to initial OCT was also recorded. Disc area and rim area were recorded for initial OCT. The relationships between CDR and disc area as well as CDR and rim area were evaluated using correlation coefficients.

**Figure 1:**
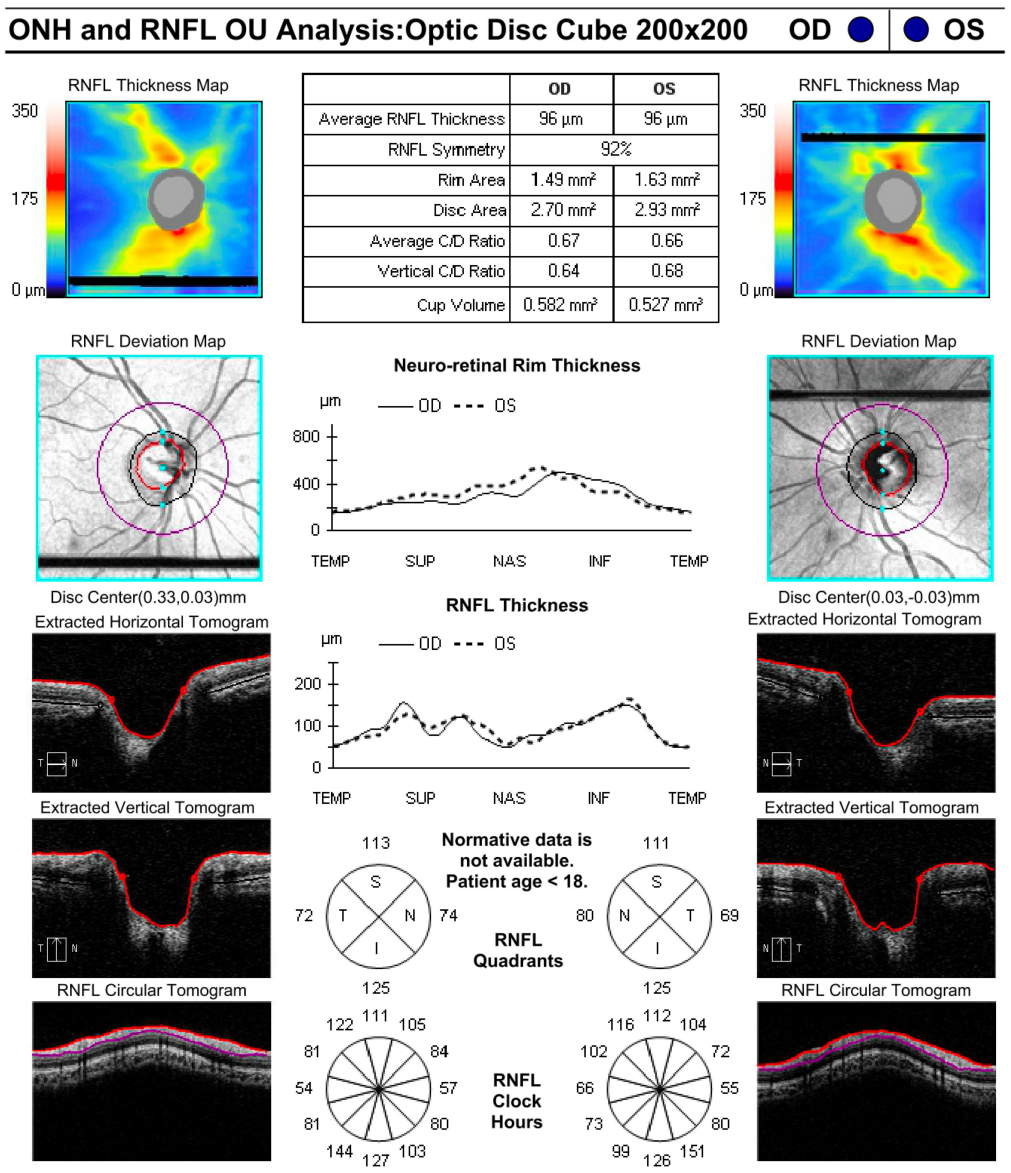
Sample quality OCT conducted on a patient 5 years and 9 months old.

During examinations, CDR was measured by clinician assessment as recorded in the electronic medical record. OCT (Zeiss Cirrus (Zeiss Meditec. Inc, Germany) was used to measure average CDR and RNFL thickness and was obtained at the discretion of the examining provider. **Figure 1** shows an example of a high-quality OCT obtained from a young patient. IOP was measured using several methods, including applanation tonometry, contact tonometry [Tonopen (Reichert, Inc., Depew, NY), and the iCare tonometer (iCare USA, Raleigh, NC)]. Measurements were obtained either in clinic or in the operating room immediately upon anesthesia induction. Measurements taken by palpation were classified as “soft” or “normal” and were not included in quantitative analysis.

At the time of final follow-up, each eye was classified as reaching one of three endpoints: continued monitoring for suspicious CDR, started on IOP-lowering drops, or conversion to glaucoma. Conversion to glaucoma was defined by CGRN criteria with at least two of the following: IOP > 21mmHg, CDR asymmetry ≥ 0.2, focal thinning of optic disc rim, presence of Haab striae, corneal edema, increased corneal diameter, axial myopia or increased axial length, VF defect, increasing CDR over time, progressive myopia, or increasing axial length unrelated to typical changes with age (3).

### Statistical analyses

Descriptive characteristics were recorded as both counts and percentages. CDRs measured by clinician assessment and OCT were compared using paired t-tests. Patients were stratified by age using 5-year increments at both time of initial assessment and time of final assessment, and CDR measured clinically and by OCT were compared within these subgroups using paired t-tests. The average difference between CDR measured by clinical assessment and OCT was measured by subtracting the clinically assessed CDR from the OCT calculated CDR for each patient and taking the mean of these values. The differences were then converted to absolute values, and the mean of the absolute values was calculated. Eyes were categorized as having either a larger CDR measured by OCT than clinical assessment, a larger CDR measured by clinical assessment than OCT, or the same CDR with both OCT and clinical assessment. CDRs measured by clinical assessment and OCT were considered the same if they were within ± 0.1 of each other. CDRs measured by clinical assessment were compared between initial and final assessment using paired t-tests. CDRs measured by OCT were also compared between initial and final assessment using paired t-tests. RNFL data were compared between initial and final OCT in patients categorized in each endpoint using t-tests. Disc area and rim area were compared among different races/ethnicities and age groups (<5 years, 5-10 years, 10-15 years, >15 years) using one-way ANOVA tests with *post hoc* Tukey tests.

All statistical analyses utilized a p-value significance threshold of p ≤ 0.05.

## Results

Two-hundred twenty-one eyes of 122 unique pediatric patients were identified and included in the present cohort. Over half of the patients were male (55%), and the average age at presentation was 9.0 years (95% CI: 8.0-9.9; **Table 1**). A majority of participants identified as white (39%) or Black/African American (39%).

**Table 1:** Demographic information of pediatric patients with suspicious CDRs.

	n
**Patients**	122
**Eyes**	221
Sex	
Male	67 (55%)
Female	55 (45%)
**Age at Presentation (years)**	9.0 ± 5.3
Race/Ethnicity	
White	47 (39%)
African American	47 (39%)
Hispanic	4 (3%)
Asian	11 (9%)
Multiracial	1 (1%)
Other	12 (10%)

Average time between initial visit and final follow-up was 5.0 years (95% CI: 4.5-5.4). **Table 2** shows ocular findings for participants on initial and final testing. Most eyes had visual acuity of better than 20/40 on initial testing (139/172, 81%) and final testing (172/211, 82%). Average refractive error (spherical equivalent) was -1.3 D (95% CI: -2–0.7) at initial visit and -2.0 D (95% CI: -3.1–0.9) at last follow-up visit. Average IOP was 16 mmHg (95% CI: 15.8-17) and 16.6 mmHg (95% CI: 15.9-17.3) on initial and final testing, respectively. Clinically measured CDR in patients who had OCT performed was not significantly different from that of the whole group on initial testing (p=0.98), but clinically measured CDR was significantly larger in patients who had OCT performed compared to that of the whole group on final testing (p=0.01).

**Table 2:** Ophthalmic findings on initial and final testing in pediatric patients with suspicious CDRs.

	Initial Visit	Final Visit
**Visual Acuity**	n=174	n=211
<20/40	139 (80%)	172 (82%)
20/40-20/70	22 (13%)	24 (11%)
20/80-20/200	9 (5%)	7 (3%)
>20/200	4 (2%)	8 (4%)
**SE (D)**	-1.3 ± 4.1	-2 ± 7.1
**IOP (mmHg)**	16 ± 3.8	16.6 ± 4.5
**CDR**	0.60 ± 0.1	0.59 ± 0.1
**CDR (clinically assessed in participants with OCT)**	0.60 ± 0.1	0.63 ± 0.1
**OCT Measurements**	n=85	n=105
Average RNFL (µm)	95.4 ± 17.4	91.5 ± 14.1
Superior RNFL (µm)	120.8 ± 21.6	114.2 ± 23.2
Inferior RNFL (µm)	118.9 ± 25	120.1 ± 23.1
Nasal RNFL (µm)	74.6 ± 21	70.1 ± 15.1
Temporal RNFL (µm)	68 ± 15.2	63.9 ± 13.4
CDR	0.65 ± 0.10	0.69 ± 0.08

**Figure 2:**
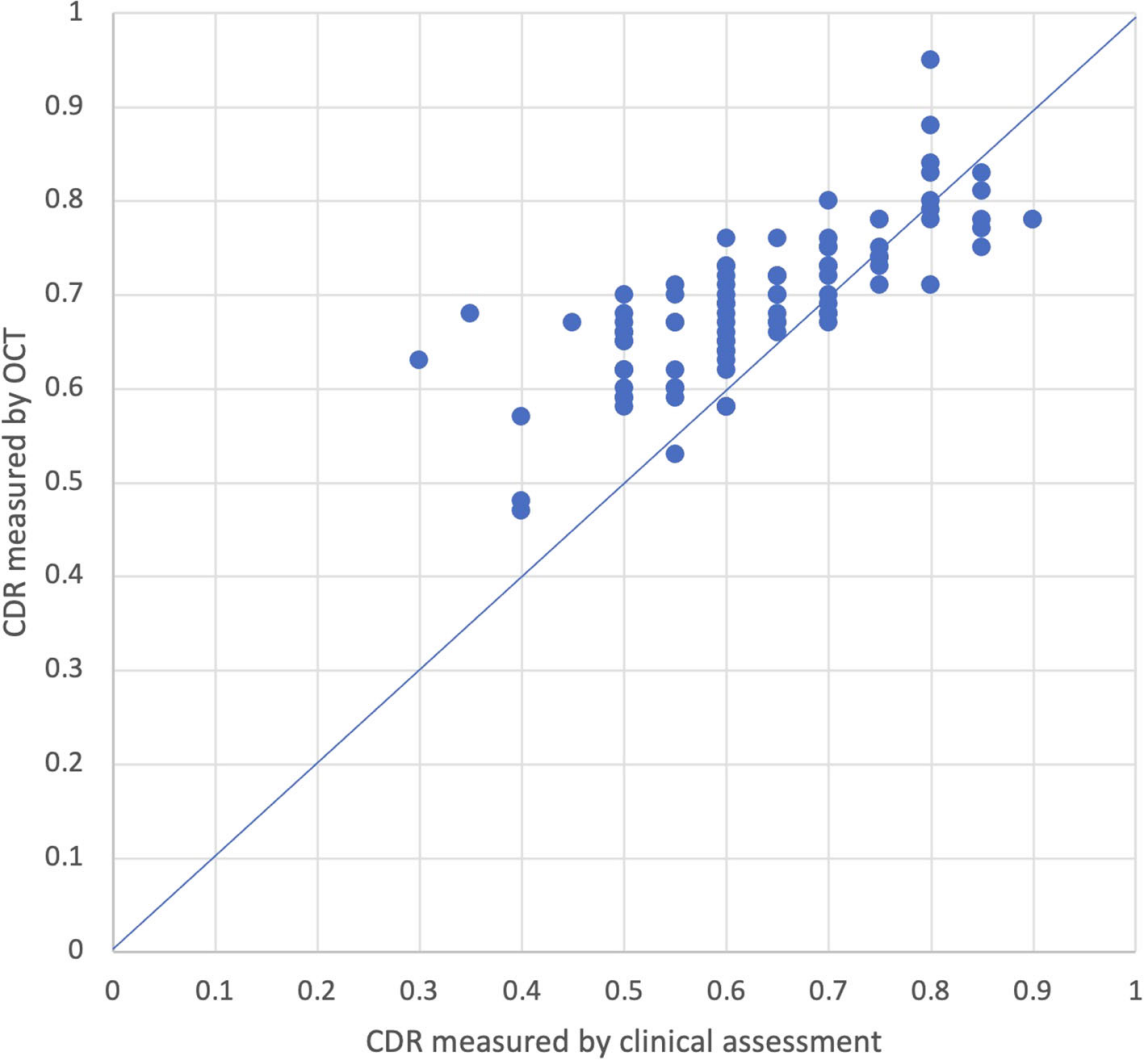
CDR measured clinically and by OCT on initial assessment (r-value = 0.25).

Fifty-three eyes had initial CDRs measured by both clinical assessment and OCT. Of these eyes, 28 had OCT CDR measurements taken on the same day as the initial visit. Among the 25 eyes that did not have measurements on the same day, the average time between measurements was 2.5 years (95% CI: 1.5-3.5). Average CDR measured by OCT (0.65; 95% CI: 0.62-0.68) was significantly larger than CDR measured by clinical assessment (0.6; 95% CI: 0.57-0.63; p=0.002; **Figure 2**). On average, CDR measured by OCT was larger than CDR measured by clinical assessment by 0.05 (95% CI: 0.02-0.08), and the absolute difference in CDR measurements between OCT and clinical assessment was 0.09 (95% CI: 0.07-0.12), with OCT measurements larger than clinical assessment. Twenty-five percent (13/53) of eyes had a larger CDR measurement with OCT compared to clinical assessment with an average difference of 0.2 (95% CI: -0.25–0.15), 6% (3/53) had a smaller CDR measurement with OCT compared to clinical assessment with an average difference of 0.23 (95% CI: 0.12-0.34), and 70% (37/53) had CDR measurements within ± 0.1. When stratified by age at time of initial visit, patients who were between 0-5 years old and 10-15 years old had CDR values that were significantly larger on OCT compared to clinical assessment (p=0.05; p=0.02) while patients between 5-10 years old and patients over 15 years old had no significant differences in CDR measured by OCT versus clinically.

Average CDR measured by initial OCT was also compared to the closest clinically measured CDR. Ninety-one percent (48/53) of eyes had measurements taken on the same day. Among the 5 eyes that did not have measurements recorded on the same day, the average time between measurements was 12.2 days (95% CI: -0.6-25). There was no significant difference between average CDR measured by OCT (0.65; 95% CI: 0.62-0.68) and average CDR measured by clinical assessment (0.63; 95% CI: 0.60-0.65). Fifteen percent (9/53) of eyes had a larger CDR measurement with OCT compared to clinical assessment with an average difference of 0.16 (95% CI: 0.14-0.19), 6% (3/53) had a smaller CDR measurement with OCT compared to clinical assessment with an average difference of 0.24 (95% CI: -0.01-0.49), and 77% (41/53) had CDR measurements within ± 0.1.

Disc area and rim area at time of first OCT were also evaluated. Average rim area was 1.30mm^2^ (95% CI: 1.25-1.35) and average disc area was 2.44mm^2^ (95% CI: 2.35-2.53). Disc area differed significantly by race/ethnicity and age (p<0.001, p<0.001). Disc area from eyes of Hispanic children was significantly larger than that of children classified as white, Asian, or other (p<0.001, p=0.01, p=0.006). Disc area in children less than 5 years old was smaller than disc area in children 5-10 years old, 10-15 years old, and >15 years old (p=0.02, p=0.002, p<0.001). Rim area also differed significantly by race/ethnicity and age (p<0.001, p=0.02). Comparison of rim area in children of different races/ethnicities was underpowered to reach significance on *post-hoc* testing. Rim area was significantly smaller in children age <5 years old compared to children 10-15 years old (p=0.002). The correlation coefficient for disc area and CDR was 0.43, and the correlation coefficient for rim area and CDR was -0.33. The ratio of disc area to CDR measured by initial OCT was compared to the ratio of disc area to CDR measured by final OCT, and no significant differences were found. Similarly, the ratio of rim area to CDR measured by initial OCT was compared to the ratio of rim area to CDR measured by final OCT, and no significant differences were found.

**Figure 3:**
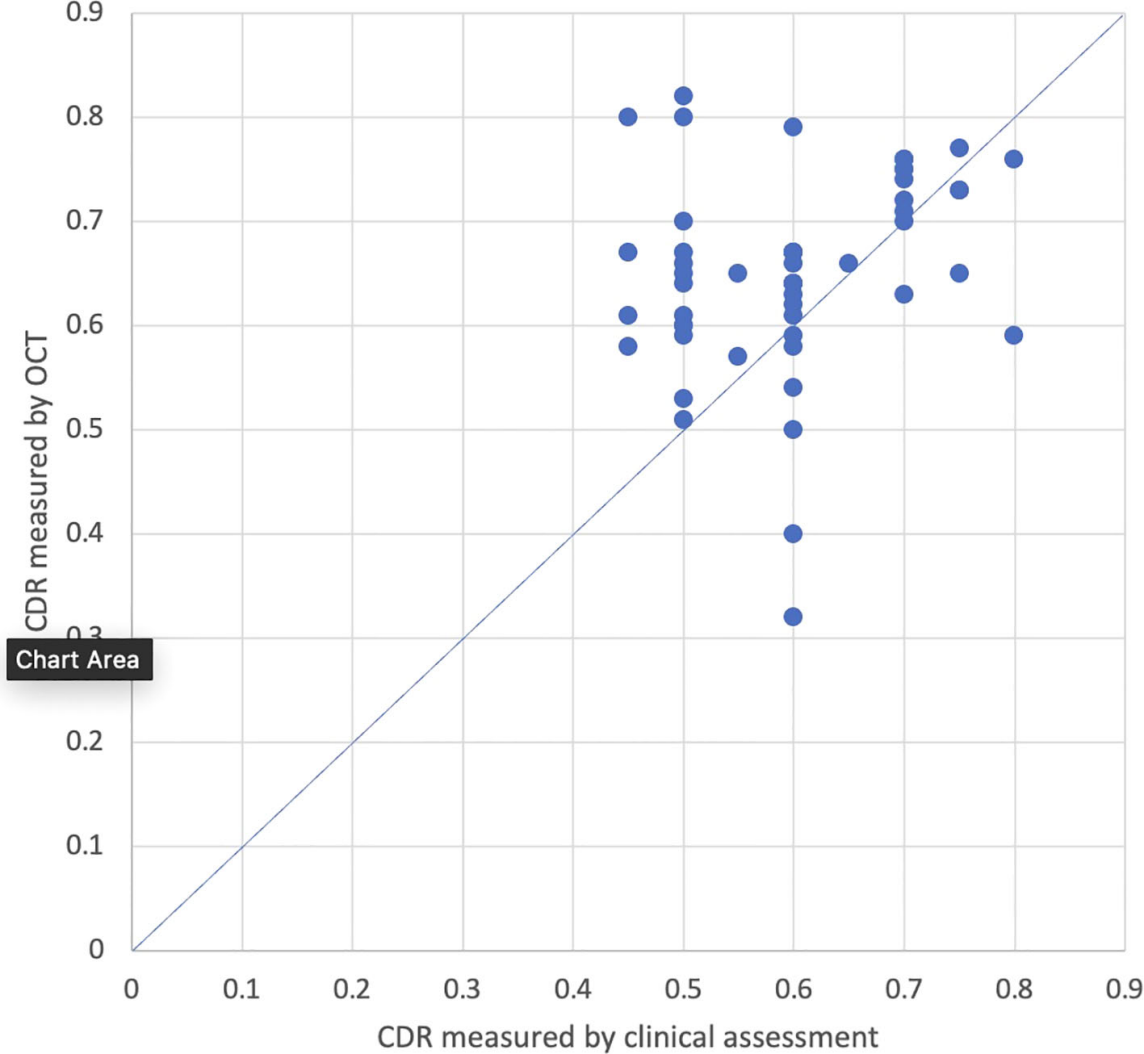
CDR measured clinically and by OCT on final follow-up (r-value = 0.77).

Ninety-three eyes had final CDR measurements conducted both clinically and by OCT, and 55 eyes had measurements taken on the same day. Among the 38 eyes that did not have measurements on the same day, the average time between measurements was 1.7 years ± 1.2 years. Average CDR measured by OCT (0.69; 95% CI: 0.68-0.71) was significantly larger than average CDR measured by clinical assessment (0.63; 95% CI: 0.61-0.66; p<0.001; **Figure 3**). The average difference between CDR measurements on final assessment was 0.06 (95% CI: 0.04-0.08). The absolute values of these differences were averaged, and the average was 0.08 (95% CI: 0.07-0.09). Twenty-seven percent (25/93) of eyes had a larger CDR measurement with OCT compared to clinical assessment with an average difference of 0.16 (95% CI: 0.14-0.19), 2% (2/93) had a smaller CDR measurement with OCT compared to clinical assessment with an average difference of 0.12 (95% CI +/-0), and 71% (66/93) had CDR measurements within ± 0.1. When stratified by age at time of final visit, patients who were between 5-10 years old, 10-15 years old, and greater than 20 years old had CDR values that were significantly larger on OCT compared to clinical assessment (p<0.001; p<0.001; p=0.01) while patients between 15-20 years old had no significant differences in CDR measured by OCT versus clinically.

On both initial testing and final follow-up, 217 eyes had CDR measured by clinical assessment and 36 eyes had CDR measured by OCT during both assessments. Average CDR measured both clinically and by OCT did not significantly differ between initial and final assessment.

### Initiation of medical therapy and conversion to glaucoma

At the time of final follow-up, nine eyes (4.1%) from six patients were started on drops to lower IOP and remained glaucoma suspects, and four eyes (1.8%) from three patients were diagnosed with glaucoma.

Brief details about the six patients who started IOP-lowering drops are described below:

1. A child being followed for enlarged CDR in both eyes with mild asymmetry who was started on topical IOP-lowering medication after developing asymmetric ocular hypertension as well as concern for interval rim thinning. OCT was recommended 6 months after initiation of drops but was not performed until several years later due to a change in eye care provider.
2. A child with a history of bilateral anterior uveitis who was on topical steroids at the time of initial presentation. The patient was noted to have increased CDR and was started on topical IOP-lowering medication with development of ocular hypertension. This patient never had OCT imaging done.
3. A child with JIA-associated anterior uveitis, complicated by cataract and started on topical IOP-lowering therapy for ocular hypertension in the setting of topical steroid use. Initial OCT was done at the time of ocular hypertension diagnosis. OCTs and HVFs were repeated initially at 3–4-month intervals and later spaced to 6–12-month intervals. These tests demonstrated consistent measurements and reassurance of no further changes in the nerve despite variable IOP measurements over time.
4. A teenager with Stickler syndrome, rhegmatogenous retinal detachment, pseudophakia, and increased CDR in one eye who was started on topical IOP-lowering medication in the setting of ocular hypertension alongside visual field changes. This patient had an OCT two years prior to initiation of drops that demonstrated thinning in the eye of concern, although repeat OCT testing was not obtained until several years later.
5. A child with neurofibromatosis, cystic fibrosis, Axenfeld-Rieger syndrome, and increased CDR bilaterally. He was started on two IOP-lowering drops in the setting of ocular hypertension, enlarged CDR, and elongated axial lengths. Baseline OCT was obtained at that time with repeat testing showing progressive RNFL change in the setting of normal IOP. This prompted lowering of target pressure and the addition of a second IOP-lowering agent. Repeat annual testing since has been stable.
6. A teenager with bilateral enlarged CDRs and bilateral anterior uveitis on topical steroids who was started on topical IOP-lowering medication after developing ocular hypertension. Baseline OCT was obtained 8 months after initiating drops and has not been obtained again since.

Brief details about the three patients who developed glaucoma are described below:

1. A child with bilateral congenital cataracts who underwent infantile cataract surgery and small sector iridectomy in the right eye. He was noted to have enlarged CDR at the time of cataract surgery with symmetrically enlarged discs (OD 2.67mm^2^, OS 2.63mm^2^), and was diagnosed with glaucoma.
2. A teenager who was initially followed for enlarged CDR who developed acute retinal necrosis and panuveitis of the right eye. He underwent lensectomy and was left aphakic, and later developed band keratopathy. He was diagnosed with glaucoma following cataract surgery in the right eye due to increased CDR and elevated IOP.
3. A young adult with a likely neurocutaneous syndrome who developed elevated IOP and increased CDR bilaterally and was diagnosed with glaucoma in both eyes. He was initially diagnosed with Sturge-Weber Syndrome as a toddler at an outside hospital based on having seizures, reported “positive” intracranial findings, and a large birthmark on the right side of his face and body. Later evaluations with Pediatric Neurology and Dermatology have suggested that his findings may be more consistent with Klippel-Trenaunay-Weber Syndrome, although he has not undergone genetic testing.

### OCT findings by endpoint

**Table 3** shows RNFL data on initial and final OCT as well as disc area and rim area on first OCT among the three endpoint groups - patients who remained glaucoma suspects, patients started on drops, and patients diagnosed with glaucoma. Those in the glaucoma subgroup demonstrated a significant decrease between initial OCT and final OCT in average RNFL thickness (p=0.01), as well as RNFL thickness in the superior and inferior quadrants (p=0.03 and p=0.02, respectively). Patients who remained glaucoma suspects and those started on drops showed no significant difference in average RNFL thickness or RNFL thickness in the superior and inferior quadrants from initial to final OCT (all comparisons with p≥0.3).

**Table 3:** RNFL data from initial and final OCTs as well as disc area and rim area measured by OCT in eyes categorized as glaucoma suspects, on drops, or diagnosed with glaucoma at the time of final follow-up.

		Glaucoma Suspects	Drops	Glaucoma`
**Average RNFL (µm)**	Initial	90.7 +/- 16.8 (n=49)	76 +/- 17 (n=2)	88.5 +/- 0.7 (n=2)
	Final	92.3 +/- 13.5 (n=88)	78.4 +/- 9.1 (n=5)	62 +/- 4.2 (n=2)
	P value	0.5	0.8	0.01*
**Inferior RNFL (µm)**	Initial	113.5 +/- 26.8 (n=49)	103.5 +/- 27.6 (n=2)	102.5 +/- 0.7 (n=2)
	Final	120.9 +/- 22.1 (n=88)	108 +/- 19.8 (n=5)	71 +/- 5.7 (n=2)
	P value	0.9	0.8	0.02*
**Superior RNFL (µm)**	Initial	118.1 +/- 22.5 (n=49)	86 +/- 17 (n=2)	118.5 +/- 4.9 (n=2)
	Final	114.4 +/- 20.9 (n=88)	96.8 +/- 16.2 (n=5)	59 +/- 14.1 (n=2)
	P value	0.3	0.5	0.03*
**Disc area (mm^2^)**		2.5 +/- 0.5 (n=106)	1.9 +/- 0.4 (n=7)	2.5 +/- 0.6 (n=4)
**Rim area (mm** ^2^)		1.3 +/- 0.1 (n=106)	1.3 +/1 0.3 (n=7)	1.4 +/- 0.3 (n=4)

Significant findings are classified as p ≤ 0.05 and are denoted with “*”.

## Discussion

Early detection of glaucoma is critical to avoid significant vision loss, especially in pediatric populations when careful examinations may be limited by patient cooperation. As increased or asymmetric CDRs may represent a potential sign of progression to glaucoma, it is important to have accurate ways of measuring CDR and monitoring change over time. This study examines the relationship between CDR measured by clinical assessment and CDR measured by OCT in pediatric glaucoma suspects. At initial and final follow-up, CDR measured by OCT was significantly larger than CDR measured by clinical assessment. This finding is consistent with similar studies in adult patients in which clinical assessment underestimated CDR compared to OCT (9, 10). On both initial and final assessment, significant differences in CDR measurements were noted more frequently in younger cohorts compared to older cohorts. This may be due to lack of cooperation during fundus examination in younger children. However, while CDR measurements differed significantly between clinical assessment and OCT, average CDR, as well as most individually matched CDR values, were within ±0.1. Thus, the difference may hold little clinical significance.

Clinical assessment of the optic nerve can be imprecise and vary among even highly trained specialists (5, 6). Pediatric patients present additional challenges to accurately measuring CDR as a majority of patients require assessment of the optic nerve head to be performed using the 20D or 28D lens, an additional percentage lack cooperation necessary to achieve a focused look at the nuances of the optic nerve head, and some patients require evaluation under anesthesia. OCT, however, can be tolerated in children as young as 3 years old, and handheld models may be used in children even younger (14). In the present study, quality OCT testing was accomplished in several young children less than 6 years old. Thus, reliably obtaining additional information about the optic nerves of patients with suspicious discs could begin as early as 5 or 6 years old in certain children. OCT computes CDR by imaging the optic nerve, measuring the ratio of the area of the cup to the area of the optic disc, and calculating the square-root of this value (16). Accurate CDR measurements are necessary for monitoring patients with suspicious optic nerves and ultimately deciding to intervene with topical or even surgical treatment. Since OCT measurements are precise and reproducible and OCT can be utilized in children, OCT may be a superior way to track CDR over time in pediatric patients being monitored as glaucoma suspects for suspicious appearance of optic discs. At present, larger CDRs suggests a need for more frequent monitoring for glaucoma (17). With a more complete picture of qualities that further increase an eye’s risk for glaucoma progression, follow-up recommendations can be even more specifically tailored. This study provides commentary on OCT in pediatric patients with increased CDR and should serve as a framework to build a greater understanding of the utility of this tool in clinical practice. It is likely in this retrospective review that patients with larger CDR underwent OCT testing more frequently to monitor the RNFL, assist in interpreting disc size, and gauge progression. While about 2/3 of patients had CDR measurements within ±0.1 between OCT and clinical evaluation, the use of images may be particularly helpful in documenting progression over time and monitoring changes in high-risk patients.

Overall, very few eyes in this cohort were started on IOP-lowering drops or converted to glaucoma. Each of these eyes had at least one finding in addition to suspicious appearance of CDR that prompted initiation of treatment or a diagnosis of glaucoma. Initiation of IOP-lowering drops was largely associated with the development of ocular hypertension, especially in the setting of topical steroid use in patients with uveitis. There was a significant amount of variability in the frequency of OCT testing in this cohort. Patients 2 and 6 were never followed by glaucoma or pediatric glaucoma specialists and received the least amount of testing while patients 3, 4, and 5 followed consistently with glaucoma or a pediatric ophthalmologist who specializes in glaucoma and had much more consistent testing. This highlights the need for a more standardized approach to testing in pediatric glaucoma suspects.

Only three participants in this study met criteria for progression to glaucoma at the time of final follow-up. Several of these eyes were initially noted to have enlarged CDR and thereafter underwent lensectomy, which may also have been a significant contributor to risk of conversion to glaucoma in these patients (18, 19). In a larger study conducted by Cardakli et al. that followed 1,375 eyes of pediatric glaucoma suspects, about 10% of eyes converted to glaucoma (15). The highest rates of conversion were seen among those being followed as glaucoma suspects for ocular hypertension (34.1%) as well as those being followed after lensectomy (16.2%). These rates are much higher than the 1.8% conversion observed in the present cohort being followed for suspicious appearance of optic discs.

RNFL data were observed at initial and final OCT for patients classified as glaucoma suspects, on drops, or diagnosed with glaucoma at the time of final follow-up. Only the group of patients with glaucoma was found to have significantly decreased RNFL on average and in the superior and inferior quadrants, supporting the diagnosis of glaucomatous-related nerve fiber layer loss as corresponding to the observed clinical changes in optic disc appearance. However, it is important to note the very small sample size of patients who ultimately developed glaucoma and were started on IOP-lowering drops, limiting comparison in these groups.

A limitation of this study is that all eyes did not receive full ophthalmologic testing at initial and final visits. While many patients had OCT testing done on the same day as initial or final assessment, other patients had varying lapses of time between clinical and OCT assessment of CDR. In addition, participant age at initial assessment varied from 6 days to 20 years, and participants were not followed for the same length of time. Mean time between initial and final assessments was also a limitation as 5 years may not be long enough to show significant changes in CDR and/or progression to glaucoma over time. Further prospective studies are needed to evaluate these findings more longitudinally. Additionally, RNFL data were compared among patients categorized into one of three endpoints, and both the group on drops and the group diagnosed with glaucoma had a very small number of members with OCT data. This size limitation makes it difficult to perform robust analyses to compare these groups and the change in RNFL over time. The small number of eyes that converted to glaucoma made it difficult to evaluate the relationship between conversion to glaucoma and optic disc measurements. To fully understand the predictive role that OCT parameters can play in following pediatric glaucoma suspects, especially those followed for suspicious optic disc, a larger, more heterogeneous pool is needed. All patients evaluated were from a single tertiary referral center, possible increasing the number of patients identified as being more at risk for developing glaucoma. Additionally, loss to follow-up of non-converter eyes as well as dismissal from the practice of low-risk eyes may potentially conflate conversion rate.

This study found that CDR measured by OCT was significantly larger than CDR measured by clinical exam. However, while the difference is statistically significant, the clinical significance is questionable. OCT, therefore, can be utilized as a method for measuring CDR in pediatric patients with suspicious optic discs overtime, especially when clinical exam proves difficult. OCT is more likely to give a conservative measurement when compared to clinical exam and is less likely to underestimate cupping. The limitations of the present study highlight the need for a prospective study that utilizes longitudinal OCT testing to evaluate changes in CDR, disc area, and rim area over time. A more robust analysis of disc area over time would help to make sense of changes in CDR, which can be unreliable on its own, especially in pre-perimetric children. Additionally, a larger cohort from a more diverse array of practices may allow for a more robust analysis of factors that are related to conversion to glaucoma. Further studies should include OCT at time of enrollment and regularly throughout the follow-up period.

## Data Availability

The raw data supporting the conclusions of this article will be made available by the authors, without undue reservation.
